# Rehabilitation outcomes of older persons within the context of the International Classification of Functioning, Disability and Health (ICF): a systematic review

**DOI:** 10.1007/s41999-026-01406-0

**Published:** 2026-01-21

**Authors:** Veerle H. E. W. Brouwer, Henk Jan Schuijt, Johanna M. A. Visser-Meily, Wilco P. Achterberg, Eléonore F. van Dam van Isselt

**Affiliations:** 1 AxionContinu, Utrecht, The Netherlands; 2 Department of Public Health and Primary Care, Leiden University Medical Center, Hippocratespad 21, 2300 RC Leiden, The Netherlands; 3 University Network for the Care Sector South-Holland, Leiden University Medical Center, Leiden, The Netherlands; 4 Center for Geriatric Trauma, St. Antonius Hospital, Utrecht, The Netherlands; 5 Internal Medicine and Geriatrics, Amsterdam UMC Location University of Amsterdam, Amsterdam, The Netherlands; 6 Department of Rehabilitation, Physical Therapy Science and Sports, UMC Utrecht Brain Center, University Medical Center Utrecht, Utrecht University, Utrecht, The Netherlands

**Keywords:** Rehabilitation, Rehabilitation for older persons, Geriatric, International Classification of Functioning Disability and Health (ICF), Rehabilitation outcomes, Biopsychosocial model

## Abstract

**Purpose:**

With the ageing population, more older persons require rehabilitation following (sub)acute functional decline. The International Classification of Functioning, Disability and Health (ICF) model offers a biopsychosocial framework to address complexities of rehabilitation in this population. This systematic review investigates which ICF components have been reported in rehabilitation research involving older persons, which rehabilitation outcomes have been reported, and whether, and how associations between ICF components and rehabilitation outcomes have been described.

**Methods:**

A comprehensive search was conducted in three databases. Studies involving older persons (mean or median age ≥ 70 years) undergoing post-acute rehabilitation, with health data linked to the ICF model and rehabilitation outcomes, were included. Study quality was assessed using the Methodological Index for Non-Randomized Studies (MINORS) by two independent reviewers.

**Results:**

Seven studies involving 896 patients were included. Associations were found between ICF components, particularly body functions and activities and participation, and improved functional independence and quality of life. Environmental factors also contributed to outcomes, whereas personal factors did not appear, reflecting their absence from the formal ICF coding structure.

**Conclusion:**

This review shows that body functions and activities and participation are most consistently linked to clinical outcomes in older persons, reflecting their central role in functional independence and quality of life. Environmental factors were examined less often, yet contextual factors (including personal factors) remain important for understanding recovery and personalized care. These findings clarify which ICF-based functional profiles are routinely captured and support more structured, person-centred assessment across the rehabilitation trajectory. Further research should include personal factors and longer-term outcomes.

**Supplementary Information:**

The online version contains supplementary material available at 10.1007/s41999-026-01406-0.

## Key summary points

**Aim:**

To investigate which ICF components have been reported in rehabilitation research involving older persons, which rehabilitation outcomes have been reported, and how associations between ICF components and rehabilitation outcomes have been described.

**Findings:**

Seven studies with 896 patients were included in a systematic review. Body functions, and activities and participation were consistently associated with functional independence and health-related quality of life. Environmental factors were also linked to rehabilitation outcomes, but personal factors were not reported because they are not coded within the ICF.

**Message:**

These findings highlight ICF components that are consistently associated with clinical outcomes in older persons and support a structured, stage-specific approach to assessment in rehabilitation research and clinical practice.

**Supplementary Information:**

The online version contains supplementary material available at 10.1007/s41999-026-01406-0.

## Introduction

The population is ageing, leading to a rise in frailty and multimorbidity, resulting in more number of older persons requiring rehabilitation after a (sub)-acute deterioration in function [1]. Multiple impairments in the physical, psychological, social, and neurocognitive domains are frequently observed in the rehabilitation population of older persons, which increases the complexity of care needs [2, 3]. Post-acute rehabilitation for older persons addresses these health problems with a multidimensional approach of diagnostic and therapeutic interventions to optimize functional capacity, promote activity and social participation, and preserve functional reserve [4].

Rehabilitation for older persons applies a holistic approach that integrates biological, psychological, and social factors, which is in line with the view of the International Classification of Functioning, Disability, and Health (ICF) model [5]. The ICF model provides a comprehensive biopsychosocial framework for understanding health and disability of an individual and their environment [5]. Through its systematic coding system, the model evaluates functioning across body structures, activities, participation, and environmental factors (Box 1). The ICF model integrates the interaction between biological and psychosocial factors and how this affects functioning, and vice versa. The ICF model could serve as a framework for capturing the complexity of rehabilitation for older persons. This suggests that the structured approach of the ICF model can help healthcare professionals understand the complex interaction between health and contextual factors, enabling them to tailor interventions to improve social participation, quality of life, and functional independence.

The ICF framework offers a standardized classification of impairments and limitations, with severity described through the use of qualifiers. An outline of components, codes, and qualifiers is provided in Box 1. The ICF components can be incorporated in rehabilitation outcome measures to evaluate changes in functional state and goal achievement [6, 7]. Functional measurement tools in rehabilitation, such as the Functional Independence Measure (FIM), the Barthel Index (BI), and the cognitive assessment Mini-Mental State Examination (MMSE) have been shown to predict rehabilitation outcomes in older persons [8–10]. However, the association between the ICF components and rehabilitation outcomes for older persons remains unknown.

The implementation of the ICF model has been described in the rehabilitation of adult populations. In 2003, Rentsch et al. described the use of the ICF model in a neurorehabilitation ward [11]. Implementation of the ICF in general neurological rehabilitation improved the quality of the interdisciplinary work process and contributed to a more systematic approach to goal assessment and discharge planning. Kus et al. [12] demonstrated how the ICF model can be used to identify factors that influence rehabilitation outcomes (return to work). Their findings highlight how the ICF model incorporates a biopsychosocial perspective, including the assessment of biological, psychological, and social factors that may impact rehabilitation outcomes.

Effective and holistic rehabilitation is essential for older persons to regain functional independence, quality of life, and social participation, in particular for the growing population with complex, multi-domain care needs. As the complexity of rehabilitation for older persons increases (driven by combinations of multimorbidity, cognitive decline, psychosocial vulnerabilities, and functional impairments), there is a growing need for using a unifying model that can integrate these dimensions in a structured and clinically meaningful way. The ICF offers a biopsychosocial framework that may support integration of multiple, complex factors by providing a structured approach to mapping functioning and contextual factors. For the ICF to serve as a clinically meaningful framework in rehabilitation for older persons, its components must be aligned with rehabilitation outcome measures commonly used in this population. This systematic review examines: (1) which ICF components have been reported in rehabilitation research involving older persons; (2) which rehabilitation outcomes have been reported; and (3) whether and how associations between ICF components and rehabilitation outcomes have been described.

Box 1: Definitions in the ICF [13] **Components**

**Fig. 1:**
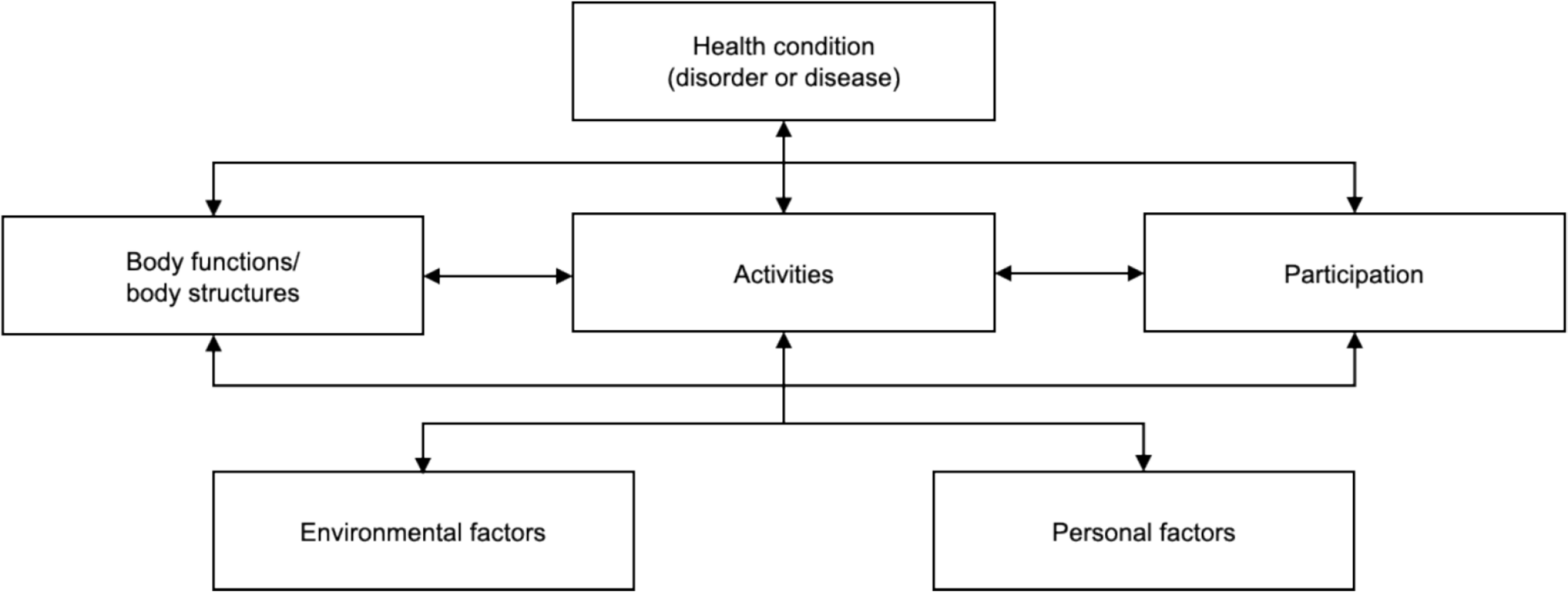
Components of the ICF model

The coding scheme of the ICF can be divided into four components, each recognizable by a letter: (s) body structures; (b) body functions; (d) activities and participation (depending on whether an activity is performed alone (activity) or in society (participation); and (e) environmental factors (Fig. 1). Although a fifth component, personal factors, is often added, this is not formally coded within the ICF model by the WHO [14].

**Interaction scheme**

The ICF components are often used as a conceptual framework for visualizing strengths and weaknesses in a person’s life, often presented as the ICF diagram (Fig. 1) [5], which helps to clarify interactions between the components and, therefore, the needs, goals, and interventions required in treatment.

**Codes**

When considering the ICF codes, the first letter represents the component (s, b, d, e); the first following number is the chapter of the component; the following numbers further specify the category level of the code. For example, code “d450” represents the component “activities and participation”, of the chapter “mobility” (4), of the category level “walking” (50). Most categories can be specified to the third level, (e.g. “d4501”, which represents “walking long distances”) and some even to a fourth level. For every category, inclusion and exclusion criteria are specified which can be found online (http://apps.who.int/classifications/icfbrowser/).

**ICF core sets**

ICF core sets assist in describing functioning, particularly in clinical practice, by providing lists of key ICF categories relevant to specific health conditions and healthcare settings. For example, in stroke populations, the comprehensive ICF core set for neurological conditions is frequently used in post-acute care. The categories of core sets are carefully selected from the full ICF model through a scientific process involving preparatory studies and input from a multidisciplinary team of experts [15].

## Materials and methods

The systematic review was reported in line with the Preferred Reporting Items for Systematic Reviews and Meta-Analyses (PRISMA) guidelines [16]. Although a protocol was developed following the PROSPERO format and submitted, it was automatically rejected due to prioritization of COVID-19-related registrations. The protocol was not altered thereafter and was followed throughout the review process. For transparency, we have added the submitted protocol in the Supplementary files (Supplement 3).

### Search strategy and selection criteria

A systematic search was conducted in EMBASE, Web of Science, and PubMed on 11 May 2024 using search terms and synonyms for ICF, aged and rehabilitation. A professional medical librarian (PG) assisted in building the search syntax (Supplement 1). No filters were applied for the search. Authors VHEWB and HJS independently screened titles and abstracts for eligibility. Studies were subjected to full-text screening if at least one author identified them as eligible. Full-text screening was independently conducted by two reviewers, and studies were included or excluded based on the predefined inclusion criteria. Conflicts were resolved through discussion to reach consensus. A third reviewer EFvDvI was consulted if the two reviewers did not reach consensus. Screening and deduplication of articles was performed in Rayyan, a systematic review management tool [17]. For all included articles, forward and backward citation tracking was conducted to identify possible additional articles.

Studies were included if they met the following predefined inclusion criteria: the population studied consisted of older persons (mean or median age ≥ 70 years) admitted for post-acute rehabilitation. Studies included data on health-related information linked to the ICF classification system (ICF-based functional profile), applied at least at the first level (chapter level d1–-d9) or higher (second-level (e.g. d4 Mobility), third-level (e.g. d450 Walking), or fourth-level categories). The studied clinical outcome was at least one of the following: length of stay (days) in the post-acute rehabilitation facility; functional independence (measured by a validated tool such as the Barthel Index (BI; [18] or the Functional Independence Measure (FIM; [19]), or a comparable measure assessed by a healthcare professional); destination after discharge (categorized as home with no services, home with services, or institutional care); disease-specific, validated patient-reported outcome measures (PROMs) that illustrate patients' experience in different health domains (including quality of life and participation); and/or rehabilitation costs. Data on clinical outcomes were gathered either at discharge or within 3e months after discharge. Study designs were either randomized controlled trials, prospective cohort studies, retrospective cohort studies, or case series, as these designs were considered most likely to provide clinically relevant information related to functioning and clinical outcomes. Studies had to be published after 2001 (the year the ICF was introduced). The full text of the article was available in English or Dutch.

### Data extraction, quality assessment, and data synthesis

Data were extracted by author VHEWB. After articles were included, a standardized data collection form was used to extract the article title, first author name, country, study design, description of the population sample, mean/median age, percentage of female participants, sample size, and the year of publication. Furthermore, author VHEWB extracted the objectives, sample size, study design, and study outcomes for each article. Study outcomes included a description of ICF-based functional profile, clinical outcomes, and the time frame in which the outcomes were collected (e.g. at admission and at discharge). ICF-based functional profile information was extracted at the most specific level reported in each study. No re-coding was applied; the synthesis remained purely narrative and descriptive, with categories interpreted in relation to their stated ICF level.

For quality assessment, the studies were rated by authors VHEWB and HJS using the Methodological Index for Non-Randomized Studies (MINORS; [20]). Data from the studies could not be pooled in a meta-analysis due to the heterogeneity of outcome measures and variability in statistical approaches across studies (t-tests, regression analyses, and correlation analyses). The synthesis of the results was a narrative review of the results, categorized by rehabilitation outcome clusters.

## Results

### Study selection

**Fig. 2:**
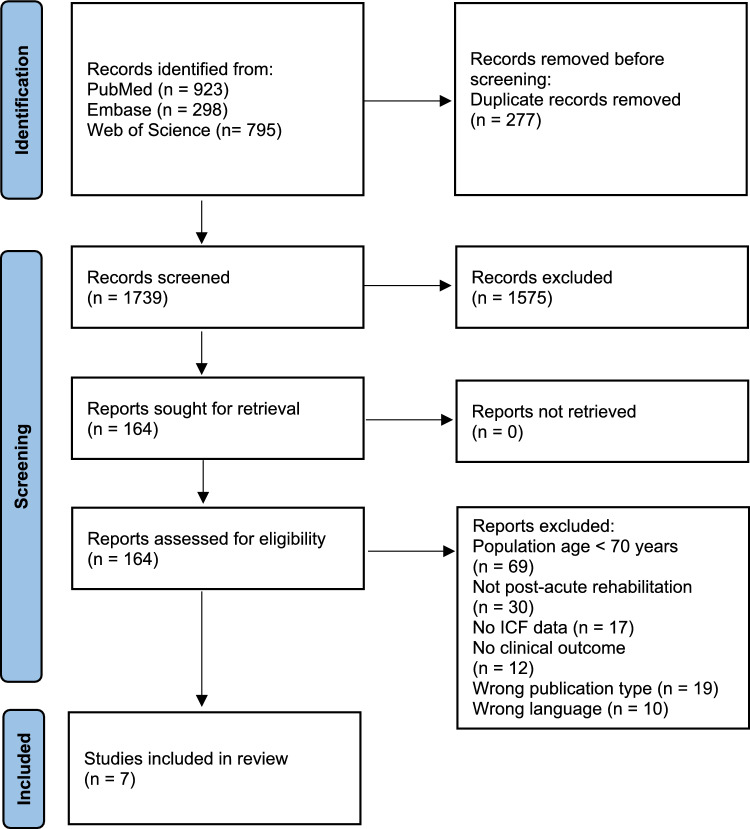
PRISMA flow diagram outlining the study selection process

A total of 2516 records were identified. After removal of duplicates and screening of titles and abstract, 164 full-text articles were reviewed, of which 7 were included in this systematic review [21–28]. No additional studies were identified by forward and backward citation tracking of the selected articles. The study selection process is illustrated in Fig. 2.

### Study characteristics

The included studies originated from Sweden, Germany, and Japan and were published between 2007 and 2020 (Table 1). Of the included studies, four were cross-sectional studies [21, 25, 26, 28], and the other three were prospective cohort studies [22, 23, 27]. Four included studies [22, 25, 27, 28] aimed to validate ICF sets and evaluate their effectiveness in post-acute and stroke rehabilitation. The other studies implemented the ICF to investigate factors associated with clinical outcomes such as quality of life [21, 23] and independent living [26].

**Table 1:** Characteristics of the included studies

First author, year	Country	Study design	Setting	Patient sample diagnosis	Sample size	Mean age in years (SD)	% Female participants
Algurèn 2012 [23]	Sweden	Prospective cohort study	Hospital stroke unit with rehabilitation	Stroke	99	72 (13.1)	54
Algurèn 2010 [28]	Sweden	Prospective cross-sectional study	Hospital stroke unit with rehabilitation	Stroke	99	72 (13.1)	54
Grill, 2007 [26]	Germany	Prospective cross-sectional study	Rehabilitation facilities	Stroke, cardiovascular, oncological, orthopaedic	128	80.3 (7.2)	69
Heise, 2016 [21]	Germany	Cross-sectional study	Geriatric care facilities	Orthopaedic	241	80.1 (7.7)	69
Kinoshita, 2016 [25]	Japan	Cross-sectional study	Stroke rehabilitation ward	Stroke	117	70.1 (14.2)	53
Kinoshita, 2020 [27]	Japan	Prospective cohort study	Stroke rehabilitation ward	Neurology and orthopaedic	104	Prior 78.2 (11.0); post 73 (13.1)^1^	Prior 60; post 53^1^
Kinoshita, 2017 [22]	Japan	Prospective cohort study	Stroke rehabilitation ward	Stroke	108	70.8 (14.0)	45.5

^1^Kinoshita et al. [27] analysed outcomes before the assessment of an ICF rehabilitation set (prior period) and after (post-period). Characteristics were reported according to the prior and post-assessment period

Sample sizes ranged from 99 [23] to 241 [21] patients, with a combined total of 896 patients. There were between 45.5% [22] and 69% [26] female patients (average 61.6% female). The mean age ranged from 70.1 [25] to 80.3 [26] years (average 75.3 years).

All studies were conducted in post-acute rehabilitation settings with a variety of patient diagnoses. Four studies included only stroke patients [22, 23, 25, 28], while the other three studies also included other diagnoses, such as orthopaedic and oncological conditions [21, 26, 27].

### Study quality and risk of bias assessment

For the MINORS quality assessment, see Table 2. As no comparative studies were included, the additional criteria for comparative studies were not assessed. All studies reported a clearly stated aim, enrolled consecutive patients, conducted prospective data collection, and used an appropriate study end point to match the aim, and used validated measures of clinical outcomes and ICF-based functional profiles. There was variability in the risk of bias associated with end point evaluation across the studies. Various methodologies were used to increase reliability, but the assessment was not fully blinded [21, 23, 25, 26]. With regard to the follow-up periods, five of the seven studies [22, 23, 26–28] reported adequate time between measurements in line with the study aim. Loss to follow-up was reported in two studies but not adequately addressed [23, 28]. Finally, two of the seven studies reported a prospective sample size calculation [23, 27], while two articles reported an ad hoc reflection on the sample size [22, 25].

**Table 2:** Study quality and risk of bias assessment for non-randomized studies (MINORS)

First author, year	Stated aim	Inclusion of consecutive patients	Prospective data collection	Appropriate end point	Unbiased evaluation of study end point	Follow-up period appropriate	Loss to follow-up < 5%	Prospective sample size calculation	Total score
Algurèn 2012 [23]	2	2	2	2	0	2	1	2	13
Algurèn 2010 [28]	2	2	2	2	1	2	1	0	12
Grill, 2007 [26]	2	2	2	2	1	2	0	0	12
Heise, 2016 [21]	2	2	2	2	1	0	0	0	9
Kinoshita, 2016 [25]	2	2	2	2	1	0	0	1	10
Kinoshita, 2020 [27]	2	2	2	2	2	2	2	2	16
Kinoshita, 2017 [22]	2	2	2	2	0	2	0	1	11

0 indicates that the criterion was not reported in the article evaluated, 1 indicates that it was reported but inadequately, and 2 indicates that it was reported adequately. The maximum score was 16 points

### Outcomes

#### Study characteristics

All included studies reported both ICF-based functional profiles and clinical outcomes. There was a variety of included ICF-based functional profiles (Table 3). None of the studies included personal factors, which reflects the structure of the ICF, as personal factors do not have official ICF codes and are therefore not included in ICF core sets or checklists.

**Table 3:** ICF-based functional profiles that were assessed in the included studies

First author, year	Body functions	Body structures	Activities	Environmental factors	Personal factors
Algurèn 2012 [23]	**✔**	**✘**	**✔**	**✔**	✘
Algurèn 2010 [28]	**✔**	✘	**✔**	✘	✘
Grill, 2007 [26]	**✔**	**✔**	**✔**	✘	✘
Heise, 2016 [21]	**✔**	✘	**✔**	**✔**	✘
Kinoshita, 2016 [25]	**✔**	**✔**	**✔**	**✔**	✘
Kinoshita, 2020 [27]	**✔**	✘	**✔**	✘	✘
Kinoshita, 2017 [22]	**✔**	**✔**	**✔**	**✔**	✘

The included studies used different ICF core sets, tailored to specific populations (Table 4). Algurèn et al. [23, 28] focused on stroke patients, assessing body functions and activities and participation from the ICF core set for stroke. The two studies reported primarily on the second-level categories of the ICF model. Grill et al. [26] used the ICF geriatric core set, also reported mainly second-level categories. Heise et al. [21] adopted a more targeted approach with three chapters from the ICF checklist, reporting on second-level categories. Kinoshita et al. [22, 25, 27] used the comprehensive ICF core set for neurological conditions and the ICF rehabilitation set for post-acute care. Across these studies [21, 22, 25–27], the ICF-based functional profiles consistently included body functions and activities and participation, all of which were reported on second-level, and occasionally third-level, categories.

**Table 4:** Study outcomes

First author, year	Clinical outcome	ICF -based functional profiles *	Data collection time points
Algurèn 2012 [23]	EQ-5D VAS	b, d, and e of the ICF core set for stroke	6 weeks, 3 months, 1 year post-stroke
Algurèn 2010 [28]	mRS	b and d of the extended ICF core set for stroke	6 weeks and 3 months post-stroke
Grill, 2007 [26]	Independence at discharge** (home with or without services vs. institutional care)	b, s, and d of the ICF geriatric core set	Discharge
Heise, 2016 [21]	EQ-5D; EQ-5D-VAS	b, d, and e codes of the ICF checklist, complemented by items specifically relevant for joint contractures	Various stages after admission
Kinoshita, 2016 [25]	FIM	b, s, d, and e from the comprehensive ICF core set for neurological conditions for post-acute care complemented by b and d of the ICF rehabilitation set	Admission and discharge
Kinoshita, 2020 [27]	FIM	b and d of the ICF rehabilitation set	Admission and discharge
Kinoshita, 2017 [22]	FIM	b, s, d, and e from the comprehensive ICF core set for neurological conditions for post-acute care complemented by b and d of the ICF rehabilitation set	Admission and discharge

*EQ-5D VAS* EuroQoL-5D visual analogue scale; *mRS* modified rankin scale; *FIM* functional independence measure; *OR* odds ratio

*For a complete and more detailed description of the categories used for each ICF-based functional profile and detailed statistical outcomes, see Supplementary Table 1

**Originally defined as determined by the ability to live independently (yes/no). b component: body functions; s component: body structures; d component: activity and participation; e component: environmental factors

Clinical outcomes were assessed using various instruments, depending on the study aims (Table 3). The EuroQoL-5D Visual Analogue Scale (EQ-5D VAS; [29]) was used to assess health-related quality of life (HRQoL) [21, 23] and the modified Rankin Scale (mRS; [30]) was used to measure perceived health status and functional independence during or after rehabilitation [21, 23, 28]. The Kinoshita et al. studies used the Functional Independence Measure [19] to assess functional independence [22, 25, 27]. Overall, data collection time points varied across the studies, with some focusing on short-term and long-term clinical outcomes (e.g. [23, 28]), one focusing on outcomes at discharge [26], and others focusing on capturing functional changes during the rehabilitation process [22, 25, 27].

To provide a framework for interpreting the distribution of ICF-based functional profiles reported across the included studies and to clarify how clinical outcomes relate to the ICF, we developed a crosswalk linking the dimensions of the clinical outcomes to ICF categories. Please refer to the crosswalk tables and further description in Supplement 2.

#### Association between ICF-based functional profiles and clinical outcomes

##### EQ-5D

Two included studies examined associations between ICF-based functional profiles and health-related quality of life (HRQoL) as measured by the EQ-5D [21, 23]. For detailed statistics of the studies, see Supplementary Table 1. Algurén et al. (2012) found that body functions were the most strongly associated with HRQoL among the ICF-based functional profiles examined [23]. Activities and participation also contributed significantly. Environmental factors did not influence HRQoL at 6 weeks. At the 1-year follow-up, body functions and environmental factors accounted for the largest proportion of variance in HRQoL outcomes. Heise et al. identified two ICF categories within the body function component, three categories within the activities and participation component, and one category within the environmental component that were significantly associated with both the EQ-5D index score and the EQ-5D VAS [21].

##### Functional independence

Five studies examined the association between ICF-based functional profiles and functional independence, primarily measured using the modified Rankin Scale (mRS) or the Functional Independence Measure (FIM) [22, 25–28]. For detailed statistics of the studies, see Supplementary Table 1. Algurén et al. (2010) examined ICF-based functional profiles associated with functional outcome, as measured by the mRS [28]. In this study, the authors identified 28 of 59 ICF categories of body functions and 41 of 59 ICF categories of activities and participation component as significantly more reported as problematic for dependent stroke survivors (i.e. mRS ≤ 2) as independent stroke survivors (i.e. mRS > 2). Grill et al. found in their development cohort that a loss of independence (defined by independence at discharge) was significantly associated with limitations in factors that are part of the body functions [26]. These associations were confirmed in a validation cohort. In a stroke population, Kinoshita et al. (2016) found a strong correlation between the FIM score and activities and participation [25]. In subsequent studies, Kinoshita et al. (2017) found significant correlations between changes in the FIM and changes in the Extension Index of the ICF rehabilitation set (number of problem categories/total number of categories × 100) of body functions and activities and participation [22]. Correlations showed moderate to strong negative associations. No significant correlations were found for body structures or environmental factors. In another study, Kinoshita et al. (2020) reported that patients who received the ICF-based multidisciplinary rehabilitation programme (post-period group) showed greater improvement in the Extension Index of the ICF rehabilitation set compared to those who did not receive the programme [27].

## Discussion

This systematic review examined which ICF-based functional profiles and clinical outcomes have been reported in rehabilitation research involving older persons, and what associations between them have been described. Seven studies were identified that reported both ICF-based functional profiles and clinical outcomes and examined the links between them. ICF-based functional profiles were evaluated using various ICF core sets, and clinical outcomes were commonly measured with established instruments for assessing functional performance in post-acute rehabilitation settings [21–23, 25–28]. Body functions and activities and participation were the most frequently reported components and were statistically associated with improved functional independence and health-related quality of life (HRQoL) in several studies [21, 23, 25, 26]. Body structures were assessed in three studies, but showed minimal change and limited associations with outcomes [22, 25, 26]. For environmental factors, two studies identified factors that contribute to improved health-related quality of life (HRQoL) and functional independence [21, 23]. Taken together, associations most consistently involved body functions and activities and participation in relation to functional independence and HRQoL, whereas evidence for body structures and environmental factors was limited. Personal factors were not addressed, consistent with their absence from the coded components of the ICF.

A key observation in this review is that body functions and activities and participation, particularly b730 (muscle power functions), d450 (walking), d455 (moving around), d510 (washing oneself) and d540 (dressing), were most frequently represented in post-acute rehabilitation for older persons and were closely linked to functional independence and HRQoL. For example, mobility codes such as d450 (walking) and self-care codes such as d540 (dressing) directly reflect FIM items and the EQ-5D mobility and self-care dimensions, which explains their prominence in the reviewed studies. This pattern is clinically plausible: body functions underpin the ability to perform mobility and self-care tasks. The crosswalk (Supplement 2) shows that commonly used clinical measures capture this same combination of body functions and activity and participation components, which likely contributes to their consistent appearance in the included studies. By contrast, cognitive functions such as b144 (memory) were reported far less often, consistent with the limited cognitive coverage of the FIM and EQ-5D. Their minimal representation in these measures likely explains their reduced appearance in the included studies.

In line with the current findings, previous studies highlight the importance of activities and participation in rehabilitation [31]. The prominence of mobility and self-care categories aligns with broader gerontology and healthy ageing frameworks. Within the WHO Healthy Ageing and intrinsic capacity models, locomotion, vitality, and the capacity to manage daily activities are described as key determinants to functional ability in older adults [32, 33]. The role of strength, endurance, and mobility as core elements of intrinsic capacity is also emphasized in the work of Bautmans [34]. These frameworks highlight the importance of mobility, muscle power, and self-care, reflecting the same components that emerged most consistently in the current review. Similar patterns have been described in ICF-based studies across rehabilitation populations, where activities and participation typically dominate functioning profiles and environmental factors are documented far less frequently [35, 36]. Content analyses of widely used outcome measures, including the FIM and EQ-5D, similarly demonstrate limited coverage of cognitive and environmental domains and a predominant focus on mobility and self-care [36, 37].

For the studies examining body structures, no significant association was found with clinical outcomes. As two out of three studies reporting body structures included stroke patients, the results are likely due to the frequently observed stability of structural impairments in the sub-acute rehabilitation phase post-stroke. Rehabilitation focuses not only on structural retraining, but above all on adaptive and compensatory strategies. A recent paper by Wade emphasizes that rehabilitation goes beyond structural recovery, serving as a catalyst for adaptation and highlighting the importance of compensatory learning in functional recovery (i.e. more focused on body functions; [38]). Adaptation, however, involves more than physical recovery and depends on personal factors such as mental resilience, coping style, and mental flexibility. Adopting person-centred approaches that involve the patient in goal setting and rehabilitation planning has been shown to improve the ability to achieve functional recovery [39].

Environmental factors, such as tailored mobility aids (e120) and support from health professionals (e355), were found to be associated with HRQoL during and after rehabilitation [21, 23]. Consistent with these findings, recent studies suggest that assistive technologies and home modifications enhance mobility, autonomy, and participation in activities of daily living among older persons; integrated rehabilitation involving multidisciplinary teams has also been shown to improve independence in ADL at discharge [40, 41]. Furthermore, social connections and engagement are closely associated with functional limitations and depressive symptoms after stroke [42].

None of the studies reported personal factors as an ICF-coded outcome, which is to be expected because personal factors are not classified within the ICF framework. Although they are not coded in the ICF framework by the WHO due to heterogeneity and contex dependence, personal factors such as coping strategies, motivation, resilience, and psychological adaptability play a critical role in how well patients can adapt to changes and manage their (changed) health conditions [31, 43, 44].

### Clinical implications

The ICF offers a framework for a systematic approach to goal setting that benefits both healthcare teams and patients [45]. Its implementation can improve care coordination, particularly in complex cases involving multiple professionals and problems in multiple domains. While the included studies mainly demonstrated associations between body functions and clinical outcomes such as HRQoL and participation, the framework itself highlights the critical role of environmental factors and personal factors. These contextual factors not only influence rehabilitation outcomes, but also provide opportunities for targeted and personalized interventions. Based on the current findings, a minimal clinically meaningful ICF set for post-acute rehabilitation for older persons may therefore focus on muscle power functions (b730), core mobility categories such as changing and maintaining body position and walking (d410–d450), and key self-care domains (d510, d540), supplemented by at least one environmental category related to support from others (e340 or e355). The current findings indicate that different ICF components are most relevant at different points in the rehabilitation trajectory. At admission, assessments may appropriately emphasize body functions and core mobility/self-care components, as limitations in these codes (b730 and d410–d450) are closely linked to early independence and change during rehabilitation [22, 25, 26, 28]. Towards discharge, broader activities and participation components gain relevance as patients regain wider functional abilities [22, 25, 27]. In the longer term, environmental influences appear increasingly important: at 1-year follow-up, environmental factors accounted for the largest variance in HRQoL outcomes, highlighting the value of capturing supports such as personal care providers (e340) and health professionals (e355) during follow-up assessments [23].

The exclusion of personal factors from the ICF coding system has been the subject of discussion. Personal factors have been described as essential for a comprehensive understanding of patient outcomes and the provision of personalized care [14, 31]. In line with Wade’s general theory of rehabilitation, understanding personal factors is essential to support patients in adjusting to health-related changes [38]. Gaining insight into factors such as health literacy, motivation, coping style, self-efficacy, and psychological resilience can provide valuable input for goal setting and treatment planning. This is particularly relevant in rehabilitation, where long-term adaptation often depends not only on functional improvement, but also on the capacity to manage change. Mapping these factors through a psychological intake or the use of questionnaires can help clinicians identify individual strengths and barriers early in the process. In research, developing and validating core sets that include personal factors (e.g. education level, coping style, gender, age) would enable healthcare professionals to better address the individualized needs of older persons. The underrepresentation of personal factors in both research and the clinical field is a pressing issue that needs addressing to advance rehabilitation research for older persons and improve personalized care strategies. For future studies, the development of ICF-based evaluation tools that integrate personal and environmental factors could have significant benefits in addressing multi-domain complexity.

### Strengths and limitations

A key strength of this review is that it brings together the reported associations between ICF domains and commonly used clinical outcome measures. By combining these findings with the conceptual overlap between ICF categories and the structure of frequently used clinical outcome measures, the review highlights a clear emphasis on body functions and activities and participation. This integrated perspective not only clarifies which aspects of functioning are most consistently measured, but also points to areas, particularly contextual factors, where opportunities remain to help guide a more person-centred rehabilitation. Another strength of this review is that the studies included are a realistic representation of the populations and settings commonly seen in rehabilitation for older persons (e.g. stroke, orthopaedic, inpatient, and hospital rehabilitation settings). This review is focused on immediate clinical outcomes, with most studies examining changes during the clinical rehabilitation period, up to discharge or three months post-discharge. This approach captures changes in functioning that occur within the structured setting of clinical rehabilitation. Of the included studies, only Alguren et al. extended follow-up to one-year post-stroke [23]. This is a limitation of the current literature, as there is a significant gap in knowledge regarding long-term functioning. Future studies could use mixed-methods approaches, incorporating patient-reported outcomes (PROMs), functional assessments, and health-related quality of life measures to provide a comprehensive understanding of long-term trajectories. This extended follow-up is particularly interesting for understanding environmental and personal factors, as the focus often transitions from the acute impact of the disease to long-term management and adaptation.

A methodological limitation is that several articles, such as that be Kool et al. [24], were excluded because they did not explicitly operationalize the ICF framework but rather used it as a general guideline. Nonetheless, these studies yielded relevant information by examining both ICF related outcomes and rehabilitation outcomes, for example by defining discharge destination (home versus not home) as an outcome measure. Although several studies found significant associations between ICF-based functional profiles and clinical outcomes, a variety of analyses and clinical measures limited comparability. As a result, no meta-analysis was possible, and findings were synthesised narratively, which reduced the ability to quantify overall effect sizes and consistency across studies. The term association is used throughout the review to denote a statistically reported link between ICF components or categories and rehabilitation outcomes, without implying causality. As a result, the review cannot establish the direction of these associations, but it does point to consistent patterns that merit exploration in future studies. Furthermore, environmental factors were not assessed in the articles that used the ICF rehabilitation core set, potentially leading to underrepresentation of this valuable component [22, 25, 27]. A more consistent use of all ICF categories in studies, together with the inclusion of environmental and personal factors, would provide a more comprehensive evaluation of clinical outcomes.

## Conclusion

This systematic review shows that body functions and activities and participation are most consistently linked to clinical outcomes in older persons, particularly muscle power, mobility and self-care in relation to functional independence and health-related quality of life. Environmental factors were reported less frequently, and personal factors did not appear because they are not included in the formal ICF coding structure. Nonetheless, both environmental and personal factors remain relevant for understanding recovery in older adults. By examining how these ICF-based functional profiles were operationalized across studies and how they relate to commonly used clinical measures, this review clarifies which aspects of functioning are routinely captured in rehabilitation for older persons and where important gaps remain. These insights inform a set of ICF categories that appear consistently relevant in this population and may serve as a pragmatic minimum reporting set for future research and clinical evaluation (i.e. a minimal data set). The findings also suggest that different ICF-based functional profiles may be particularly informative at different stages of rehabilitation, supporting a more structured and person-centred approach to assessment across the rehabilitation trajectory.

## Supplementary Information

Below is the link to the electronic supplementary material.

Supplementary file1 (DOCX 56 KB)

## Data Availability

No new data were created or analyzed in this study. The data extraction form and related materials used during the current study are available from the corresponding author upon reasonable request.

## Abbreviations

ADL: Activities of daily living
BI: Barthel index
EQ-5D: EuroQol 5D
FIM: Functional independence measure
HRQoL: Health-related quality of life
ICF: International classification of functioning disability and health
mRS: Modified rankin scale
MMSE: Mini-mental state examination
OR: Odds ratio
PROM: Patient-reported outcome measure
VAS: Visual analogue scale
