# Supplementary material for: Rehabilitation outcomes of older persons within the context of the International Classification of Functioning, Disability and Health (ICF): a systematic review
Source: Eur Geriatr Med. 2026 Jan 21;17(1):261–72. doi: 10.1007/s41999-026-01406-0 (PMC12945888; doi:10.1007/s41999-026-01406-0)
Supplement: Supplementary file 1 — Supplementary file1 (DOCX 56 KB) [file 41999_2026_1406_MOESM1_ESM.docx]

**Supplementary files**

**Title:** Rehabilitation outcomes of older persons within the context of the International Classification of Functioning, Disability and Health (ICF): a systematic review.

**Authors:** V.H.E.W. Brouwer^1,2,3^, H.J. Schuijt^4,5^, J.M.A. Visser-Meily^6^, W.P. Achterberg^2,3^, E.F. van Dam van Isselt^2,3^

^1^AxionContinu, Utrecht, The Netherlands

^2^Department of Public Health and Primary Care, Leiden University Medical Center, Leiden, The Netherlands

^3^University Network for the Care sector South-Holland, Leiden University Medical Center, Leiden, The Netherlands

^4^Center for Geriatric Trauma, St. Antonius Hospital, Utrecht, The Netherlands

^5^Internal Medicine and Geriatrics, Amsterdam UMC Location University of Amsterdam, Amsterdam, The Netherlands

^6^Department of Rehabilitation, Physical Therapy Science and Sports, UMC Utrecht Brain Center, University Medical Center Utrecht, Utrecht University, Utrecht, The Netherlands

**Corresponding author:** V.H.E.W. Brouwer, Hippocratespad 21, 2300 RC Leiden, The Netherlands

Email: [v.h.e.w.brouwer@lumc.nl](mailto:v.h.e.w.brouwer@lumc.nl)

## Supplement 1. Search strings for Pubmed, Embase and Web of Science

*Pubmed*

("International Classification of Functioning, Disability and Health"[Mesh] OR "International

Classification of Functioning Disability and Health"[tw] OR "ICF"[tw] OR "functioning and

disabilit*"[tw] OR "International classification of function*"[tw] OR "disability function*"[tw])

AND (("Aged"[Mesh] OR "Aged, 80 and over"[Mesh] OR "frail elderly"[MeSH] OR "elderly"[tw]

OR "elder"[tw] OR "elders"[tw] OR "aged"[ti] OR "aging"[tiab] OR "ageing"[tiab] OR "oldest

old"[tw] OR "older person*"[tw] OR "old person*"[tw] OR "older patient*"[tw] OR "old

patient*"[tw] OR "older women"[tw] OR "old women"[tw] OR "older men"[tw] OR "old men"[tw]

OR "old adult*"[tw] OR "older adult*"[tw] OR "Older individual*"[tw] OR "old people"[tw] OR

"older people"[tw] OR "septuagenarian*"[tw] OR "octagenarian*"[tw] OR "octogenarian*"[tw] OR

"nonagenarian*"[tw] OR "centenarian*"[tw] OR "senescence"[tw] OR "senescent"[tw] OR

"geriatric"[tw] OR "geriatrics"[tw] OR "geriatrics"[MeSH] OR "older"[ti] OR "senior"[tw] OR

"seniors"[tw] OR "older population"[tw]) AND ("Rehabilitation"[Mesh] OR "rehabilitation"

[Subheading] OR "Habilitation"[tw] OR "Rehab*"[tw])

Limits: 01-01-2001 till 05-11-2024

*Embase*

(exp "International Classification of Functioning, Disability and Health"/ or "International

Classification of Functioning Disability and Health".tw. or "ICF".tw. or "functioning and

disabilit*".tw. or "International classification of function*".tw. or "disability function*".tw.) and (exp

aged/ or exp "Aged, 80 and over"/ or exp "frail elderly"/ or elderly.tw. or elder.tw. or elders.tw. or

aged.ti. or aging.ti,ab. or ageing.ti,ab. or oldest old.tw. or older person*.tw. or old person*.tw. or older

patient*.tw. or old patient*.tw. or older women.tw. or old women.tw. or older men.tw. or old men.tw.

or old adult*.tw. or older adult*.tw. or Older individual*.tw. or old people.tw. or older people.tw. or

septuagenarian*.tw. or octogenarian*.tw. or nonagenarian*.tw. or centenarian*.tw. or senescence.tw. or senescent.tw. or geriatric.tw. or geriatrics.tw. or geriatrics/ or older.ti. or senior.tw. or seniors.tw. or

older population.tw.) and (exp "Rehabilitation"/ or "rehabilitation".tw. or "Habilitation".tw.)

Limits: 01-01-2001 till 05-11-2024

*Web of Science*

TS=("International Classification of Functioning Disability and Health" OR "ICF" OR "functioning

and disabilit*" OR "International classification of function*" OR "disability function*") AND

TS=("Aged" OR "Aged, 80 and over" OR "frail elderly" OR elderly OR elder OR elders OR aged OR

aging OR ageing OR oldest old OR older person* OR old person* OR older patient* OR old patient*

OR older women OR old women OR older men OR old men OR old adult* OR older adult* OR Older

individual* OR old people OR older people OR septuagenarian* OR octogenarian* OR nonagenarian*

OR centenarian* OR senescence OR senescent OR geriatric OR geriatrics OR geriatrics OR older OR

senior OR seniors OR older population) AND TS=("Rehabilitation" OR "rehabilitation" [Subheading]

OR "Habilitation")

Limits: 01-01-2001 till 05-11-2024

## Supplementary Table 1. Detailed study outcomes.

| **First author, year** | **Clinical outcome** | **ICF-based functional profile** | **Statistical analysis** | **Main statistical findings** |
| --- | --- | --- | --- | --- |
| Algurèn 2012 [23] | EQ-5D VAS | b codes (59 categories), d codes (59 categories), e codes (37 categories) of the ICF core set for stroke | Multivariate regression at 3 timepoints | At 6 weeks: b (β = 0.46, p < .001, R² = 0.93), d (β = 0.39, p < .001, R² = 0.80), e not significant  At 3 months: b (β = 0.41, p < .001, R² = 0.89), d (β = 0.38, p < .001, R² = 0.84), e not significant  At 1 year: e (codes e580 and e540) were significant predictors of HRQoL (R² = 0.52), b, d not significant. |
| Algurèn 2010 [28] | mRS | b codes (59 categories), d codes (59 categories) of the extended ICF core set for stroke | Descriptive analysis and non-parametric group comparison | Dependent survivors (mRS > 2) compared to independent survivors (mRS ≤ 2) more often reported problems with mobility (d4; M_diff_ = 4.8, p < .001), self-care (d5; M_diff_ = 2.9, p < .001), neuromusculoskeletal and movement-related functions (b7; M_diff_ = 2.6, p < .01), genitourinary and reproductive functions (b6; M_diff_ = 0.3, p < .05), and communication (d3; M_diff_ = 1.6, p < .01) at six weeks and three months. |
| Grill, 2007 [26] | Independence at discharge* (home with or without services vs. institutional care) | b codes (45 categories), s codes (12 categories), d codes (15 categories) of the ICF geriatric core set | Multivariate logistic regression  in a development and validation cohort | Development cohort: moving around using equipment (d465) (OR = 2.7, 95% CI 1.2–5.8) and clinical indicators for fracture/joint replacement (OR = 2.3, 95% CI 1.1–4.9). Validation cohort: d465 (OR = 7.6, 95% CI 1.6–35.5), b765 (OR = 5.9, 95% CI 2.6–13.4). |
| Heise, 2011 [21] | EQ-5D; EQ-5D-VAS | Body functions (b codes; 21 categories), activities and participation (d codes; 23 categories), environmental factors (e codes; 15 categories) of the ICF checklist, complemented by items specifically relevant for joint contractures | Multiple linear regression | Muscle power functions (b730) (β = -0.08; p = 0.02), taking care of plants (d6505) (β = -0.06; p = 0.03), recreation and leisure (d920) (β = -0.09; p = 0.03), and drugs (e1101) (β = 0.07; p = 0.03). R² = 0.28 for EQ-5D; R² = 0.26 for EQ-5D-VAS. |
| Kinoshita, 2016 [25] | FIM | b codes (54 categories), s codes (11 categories), d codes (34 categories), e codes (17 categories) from the comprehensive ICF core set for neurological conditions for post-acute care complemented by b codes (9 categories), d codes (21 categories) of the ICF rehabilitation set | Spearman’s rank-order correlation analysis | FIM total with d (*r* = –.86 / –.76), motor FIM with d4–5 (*r* = –.85 / –.79), cognitive FIM with d1–3 (*r* = –.85 / –.74), and weak correlations for participation with FIM total score and all FIM subscales (d6–9; *r* = –.27 to –.44); all *p* < .001. No significant correlations for b, s. |
| Kinoshita, 2020 [27] | FIM | b codes (8 categories), d codes (15 categories) of the ICF rehabilitation set | Multiple linear regression | Greater improvement in Extension Index in post-period group vs. prior-period: 31.6 vs. 17.3, 95% CI 7.0–21.5, p < .001; effect remained after adjustment age, sex, disease type, and baseline score (β = 8.5, 95% CI 1.7–15.3, p = .014); no significant differences in FIM admission, discharge, or gain. |
| Kinoshita, 2017 [22] | FIM | b codes (54 categories), s codes (11 categories), d codes (34 categories), e codes (17 categories) from the comprehensive ICF core set for neurological conditions for post-acute care complemented by b codes (9 categories), d codes (21 categories) of the ICF rehabilitation set | Spearman’s rank-order correlation analysis | *r* = –.36 to –.21 (b), –.46 to –.45 (d); all *p* < .05; no significant correlations for s or e |

*Notes.* * Originally defined as determined by the ability to live independently (yes/no). b: body functions; s: body structures; d: activity and participation; e: environmental factors. EQ-5D VAS: EuroQoL-5D Visual Analogue Scale; mRS: modified Rankin Scale; FIM: Functional Independence Measure; OR: Odds Ratio.

## Supplement 2. ICF-based crosswalk between clinical outcome measures and ICF categories

To support the interpretation of how the included outcome measures relate to the ICF, crosswalks were developed for the FIM, EQ-5D and mRS. Using Cieza’s linking rules, we mapped each item or dimension of the clinical outcome measure to the most specific available ICF category (3rd/4th level where applicable) [1,2]. These mappings formed the basis for interpreting the distribution of ICF categories in the included studies. Where published ICF mappings were available, these were used directly; where they were not, the main principles of the linking rules were applied to identify the most appropriate categories.

*2.1. FIM*

To clarify how the FIM corresponds to aspects of functioning, each motor and cognitive item was linked to the most relevant ICF categories (Supplementary Table 2). Where published ICF mappings existed, these were adopted directly, including studies comparing the content of the FIM with ICF Core Sets and detailed item-level mappings [3, 4]. The resulting crosswalk shows that the FIM primarily reflects activities and participation, especially self-care and mobility (d5 and d4), with a smaller subset of body function categories related to continence, communication and higher-level cognitive functions. Environmental factors are not represented.

*Supplementary Table 2.* Conceptual crosswalk between all FIM items and ICF-based functional profiles.

| **FIM item** | **Main ICF component(s)** | **2nd-level ICF category** | **3rd-level codes (examples)** |
| --- | --- | --- | --- |
| 1. Eating | d Activities & participation | d550 Eating | d5500 Eating; d5501 Drinking |
| 1. Grooming | d Activities & participation | d520 Caring for body parts | d5200 Caring for skin; d5202 Caring for hair |
| 1. Bathing | d Activities & participation | d510 Washing oneself | d5100 Washing body parts; d5101 Washing whole body |
| 1. Dressing – Upper body | d Activities & participation | d540 Dressing | d5400 Putting on clothes |
| 1. Dressing – Lower body | d Activities & participation | d540 Dressing | d5400 Putting on clothes |
| 1. Toileting | d Activities & participation | d530 Toileting | d5300 Regulating urination; d5301 Regulating defecation |
| 1. Bladder management | b Body functions; d Activities | b620 Urination functions; d530 Toileting | b6202 Urinary continence |
| 1. Bowel management | b Body functions; d Activities | b525 Defecation functions; d530 Toileting | b5252 Faecal continence |
| 1. Bed/Chair/Wheelchair transfer | d Activities & participation | d410 Changing body position; d420 Transferring oneself | d4100 Lying down; d4200 Transferring while sitting |
| 1. Toilet transfer | d Activities & participation | d410; d420 | d4201 Transferring while standing |
| 1. Bath/Shower transfer | d Activities & participation | d410; d420 | d4103 Sitting; d4200 Transferring oneself |
| 1. Walking/Wheelchair | d Activities & participation | d450 Walking; d455 Moving around | d4500 Walking short distances; d4551 Walking long distances |
| 1. Stairs | d Activities & participation | d455 Moving around | d4551 Climbing stairs |
| 1. Comprehension | d Activities & participation | d310 Communicating – receiving messages | d3100 Receiving spoken messages |
| 1. Expression | d Activities & participation | d330 Speaking | d3300 Producing speech |
| 1. Social interaction | d Activities & participation | d710 Basic interpersonal interactions | d7100 Initiating interactions |
| 1. Problem solving | b Body functions | b164 Higher-level cognitive functions | b1641 Organisation and planning; b1646 Problem-solving |
| 1. Memory | b Body functions | b144 Memory functions | b1440 Short-term memory; b1441 Long-term memory |

*2.2.* EQ-5D

To clarify how the EQ-5D dimensions relate to functioning, each dimension was linked to the most relevant ICF categories (Supplementary Table 3). Where published ICF-based comparisons of health-related quality of life instruments were available, these were used directly to inform the mapping [5]. The resulting crosswalk shows that the EQ-5D mainly captures mobility, self-care and general daily activities, with limited representation of body functions and no explicit environmental categories.

*Supplementary Table 3*. Conceptual crosswalk between EQ-5D dimensions and ICF categories

| **EQ-5D dimension** | **Main ICF component(s)** | **2nd-level ICF category** | **3rd-level codes (examples)** |
| --- | --- | --- | --- |
| Mobility | d Activities & participation | d450 Walking; d455 Moving around | d4500 Walking short distances; d4551 Climbing stairs |
| Self-care | d Activities & participation | d510 Washing; d520 Caring for body parts; d540 Dressing; d550 Eating | d5100 Washing body parts; d5400 Putting on clothes |
| Usual activities | d Activities & participation | d230 Daily routine; d440 Fine hand use; d570 Looking after one’s health |  |
| Pain/Discomfort | b Body functions | b280 Sensation of pain | b2801 Pain in body part; b2804 Generalized pain |
| Anxiety/Depression | b Body functions | b152 Emotional functions | b1520 Appropriateness of emotion |

2.3. mRS

To clarify how the mRS relates to functioning, we developed a structured decomposition informed by published ICF-linking studies (Supplementary Table 4). Although the mRS is not item-based, its descriptors follow recognizable patterns: scores 1–2 reflect mild limitations in daily routine and mobility; scores 3–4 denote increasing dependence in basic activities of daily living; and score 5 represents global dependency, often requiring environmental support. These interpretations are consistent with previous ICF-linking work showing that the mRS spans several activity, participation and body function domains but lacks the specificity needed for precise category assignment [6, 7, 8].

*Supplementary Table 4*. Structured decomposition of the modified Rankin Scale and ICF-based functional profiles.

| mRS score | Functional description | ICF-based functional profiles involved |
| --- | --- | --- |
| 0 | No symptoms | None |
| 1 | Symptoms without functional limitations | d Activities and participation |
| 2 | Slight disability; independent in personal care | d Activities & Participation; b Body functions |
| 3 | Moderate disability: assistance required but ambulant | d Activities & Participation; b Body functions |
| 4 | Moderately severe disability; dependent for mobility and bodily care | d Activities & Participation; b Body functions; e Environmental factors |
| 5 | Severe disability; fully dependent with continuous care needs | d Activities & Participation; b Body functions; e Environmental factors |
| 6 | Death | Not applicable |

## Supplement 3. Submitted PROSPERO Protocol (not registered)

## Review title

The International Classification of Functioning, Disability and Health (ICF) model as a predictor for geriatric rehabilitation outcomes: a systematic review.

## Anticipated start date

01 May 2024

## Anticipated completion date

31 October 2024

## Stage of review at submission

The review had not yet started at the time of submission.

## Named contact

V.H.E.W. Brouwer MSc (Leiden University Medical Center, The Netherlands)

## Funding

None.

## Conflicts of interest

None declared.

## Review question

Do the International Classification of Functioning, Disability and Health (ICF) qualifiers predict rehabilitation outcomes in geriatric patients?

## Databases searched

Embase, PubMed, and Web of Science.

URL search strategy for PubMed: <https://www.crd.york.ac.uk/PROSPEROFILES/541833_STRATEGY_20240430.pdf>

## Condition / domain

Rehabilitation medicine.

## Population

Older individuals (mean age ≥70 years) admitted for post-acute inpatient rehabilitation.

## Intervention/ exposure

Use of the International Classification of Functioning, Disability and Health (ICF) model.

## Comparator

Not applicable.

## Types of studies to be included

1. Studies include older individuals (mean or median age ≥70 years) admitted for post-acute inpatient rehabilitation.
2. Data on health-related information linked to the International Classification of Functioning, Disability and Health (ICF) classification system, at least at the first level (chapter level d1–d9) of classification or higher.
3. The rehabilitation outcome is at least one of the following: - Length of stay (days)
   1. Functional independence defined by the Barthel Index (BI) or the Functional Independence Measure (FIM), or comparable validated measure assessed by a healthcare professional
   2. Destination after discharge; categorized as home with no services, home with service, or institutional care
   3. Rehabilitation costs
   4. Disease-specific validated patient-reported outcomes (PROMs)
4. The rehabilitation outcome data originates at the time of discharge or at least within three months after discharge
5. Randomized controlled trial, prospective cohort studies, retrospective cohort studies, case series VI) Published after 2001
6. Full text in English/Dutch

## Context

Further elaboration on disease-specific validated patient-reported outcomes (PROMs) that illustrate the experience of the patients in different health domains:

1) Quality of life (measured by the generic QoL questionnaires such as the SF-12, SF-36, EQ-5D, WHOQOL- BREF or disease-specific QoL questionnaire such as the SS-QOL)

2) Mental health: depression and anxiety symptom measures

3) Participation (WHODAS; P-scale, or other validated participation measure)

## Main outcomes

The predictive value of the ICF in geriatric rehabilitation.

- Length of stay (days);
- Functional independence, defined by the Barthel Index (BI) or the Functional Independence Measure (FIM), or a comparable validated measure assessed by a healthcare professional;
- Destination after discharge, categorised as home without services, home with services, or institutional care;
- Rehabilitation costs;
- Disease-specific validated patient-reported outcomes (PROMs) reflecting patients’ experiences across different health domains, including:
  - Quality of life, measured using generic questionnaires (e.g. SF-12, SF-36, EQ-5D, WHOQOL-BREF) or disease-specific instruments (e.g. SS-QOL);
  - Mental health, assessed by measures of depressive and anxiety symptoms;
  - Participation, measured using instruments such as the WHODAS, P-scale, or other validated participation measures.

## Data extraction

Data will be extracted from three databases by one reviewer (HEWB). Two reviewers (HJS and HEWB) will independently assess titles and abstracts for eligibility. Full-text screening will be performed when at least one reviewer considers a study eligible. In case of disagreement, a third reviewer (EFvD) will be consulted to reach consensus. Screening of articles will be conducted using Rayyan after deduplication in Mendeley. Following study inclusion, a standardised data extraction form will be used to collect information on article title, first author, country, study design, population characteristics, mean or median age, proportion of female participants, sample size, database of retrieval, year of publication, and date of search. In addition, reviewers will extract study objectives and outcomes using a separate form, distinguishing between ICF-related characteristics and rehabilitation outcomes. Forward and backward citation tracking will be performed for all included articles to identify additional relevant studies.

## Risk of bias assessment

For quality assessment, the studies are rated using the Methodological Index for Non-Randomized Studies (MINORS) for cohort studies and the Cochrane risk of Bias tool for randomized controlled trials.

## Data synthesis

The principal approach to data synthesis is a narrative review of the results.

## Type of review

Systematic review.

## Health areas of the review

Care of the elderly; Rehabilitation.

## Country

The Netherlands.

## Planned dissemination

Publication in an international peer-reviewed journal.

**Supplementary files references**

1. Cieza A., Brockow T., Ewert T., Amman E., Kollerits B., Chatterji S., Üstün T. B., & Stucki G. (2002). Linking health-status measurements to the International Classification of Functioning, Disability and Health (ICF). Journal of Rehabilitation Medicine, 34, 205–210.
2. Cieza A., Fayed N., Bickenbach J., & Prodinger B. (2019). Refinements of the ICF Linking Rules to strengthen their potential for establishing comparability of health information. Disability and Rehabilitation, 41(5), 574–583.
3. Schepers, V. P. M., Ketelaar, M., van de Port, I. G. L., Visser-Meily, J. M. A., & Lindeman, E. (2007). Comparing contents of functional outcome measures in stroke rehabilitation using the International Classification of Functioning, Disability and Health (ICF). Disability and Rehabilitation, 29(3), 221–230.
4. Fréz, A. R., Nascimento, L. R., Ferreira, S., Scianni, A. A., & Teixeira-Salmela, L. F. (2013).

Relationship between the Functional Independence Measure and the International Classification of Functioning, Disability and Health Core Set for stroke. Acta Fisiátrica, 20(3), 135–140.

1. Cieza, A., & Stucki, G. (2005). Content comparison of health-related quality of life (HRQOL) instruments based on the International Classification of Functioning, Disability and Health (ICF). Quality of Life Research, 14(5), 1225–1237.
2. Berzina, G., Vetra, A., Krievins, D., & Smane, E. (2016). Linking the Modified Rankin Scale to the International Classification of Functioning, Disability and Health. Journal of Rehabilitation Medicine, 48(3), 261–267.
3. Silva, S. M., Ribeiro, O., Santos, C., & Monteiro, A. (2023). Mapping the Modified Rankin Scale to the ICF: A conceptual examination of disability levels after stroke. International Journal of Environmental Research and Public Health, 20(8), 1277.
4. Berzina, G., Sveen, U., Paanalahti, M., & Sunnerhagen, K. S. (2016). Analyzing the modified Rankin Scale using concepts of the International Classification of Functioning, Disability and Health. European Journal of Physical and Rehabilitation Medicine, 52(2), 203–213. https://doi.org/10.3109/09638288.2016.1145258
